# Protein polymers proliferate: polymerization of phosphatases and proteases

**DOI:** 10.1128/spectrum.02439-26

**Published:** 2026-09-14

**Authors:** Ravi R. Sonani, Iman Chouikha, Thijs Bodson, Silvia Hidalgo-Martinez, Filip J. R. Meysman, Han Remaut, Geneviève Ball, Edward H. Egelman, Romé Voulhoux, Mike Sleutel

**Affiliations:** 1 Department of Biochemistry and Molecular Genetics, University of Virginia Medical School 12349, Charlottesville, Virginia, USA; 2 CNRS, Aix Marseille Université, LCB-UMR7283, IM2B, Marseille, France; 3 Structural Biology Brussels, Department for Bio-engineering Sciences, Vrije Universiteit Brussel 70493, Brussels, Belgium; 4 Structural and Molecular Microbiology, Vlaams Instituut voor Biotechnology (VIB) Center for Structural Biology, Vrije Universiteit Brussels 70493, Brussels, Belgium; 5 Department of Biology, University of Antwerp 98888, Antwerp, Belgium; The Pennsylvania State University, University Park, Pennsylvania, USA

**Keywords:** cryo-EM, enzymes, polymers

## Abstract

It has been known for more than half a century that enzymes can polymerize to either activate or repress their catalytic functions. Using electron cryo-microscopy (cryo-EM), we serendipitously found that two commonly used enzymes in molecular microbiology research, alkaline phosphatase and proteinase K, can unexpectedly form filaments. While the physiological significance of fibrillation in alkaline phosphatase and proteinase K is unknown, the atomic models of these polymers show the ability of cryo-EM to discover and determine high-resolution structures of macromolecular assemblies before they have been characterized or purified.

**IMPORTANCE:**

Electron cryo-microscopy (cryo-EM) has emerged over the past decade as the dominant technique in structural biology for determining the atomic structures of biological macromolecular complexes. One less appreciated feature of cryo-EM is that, in many cases, complexes do not need to be purified. Thus, the atomic structures of assemblies that can be found in biological samples as “contaminants” can be readily determined. In this paper, the structures of filaments formed by a phosphatase and a protease are described.

## OBSERVATION

Since acetyl coenzyme A carboxylase was discovered to form filaments more than 55 years ago (1), the literature on enzyme polymerization has grown considerably (2–5). Filamentation has been abundantly documented to be responsible for either enzyme activation (6) or repression (7), and thus the polymeric state of an enzyme can provide a new level of biological regulation. In the course of examining bacterial samples by electron cryo-microscopy (cryo-EM), we have serendipitously discovered a previously unreported filamentation of two commonly used enzymes in molecular microbiology: alkaline phosphatase (ALP) and proteinase K (ProtK). This report describes the structure of these enzyme polymers at near-atomic resolution and highlights the increasing ability of using cryo-EM to determine high-resolution structures of macromolecular assemblies before they have been characterized or purified (8).

### The scaffolded ALP filament

**Fig 1:**
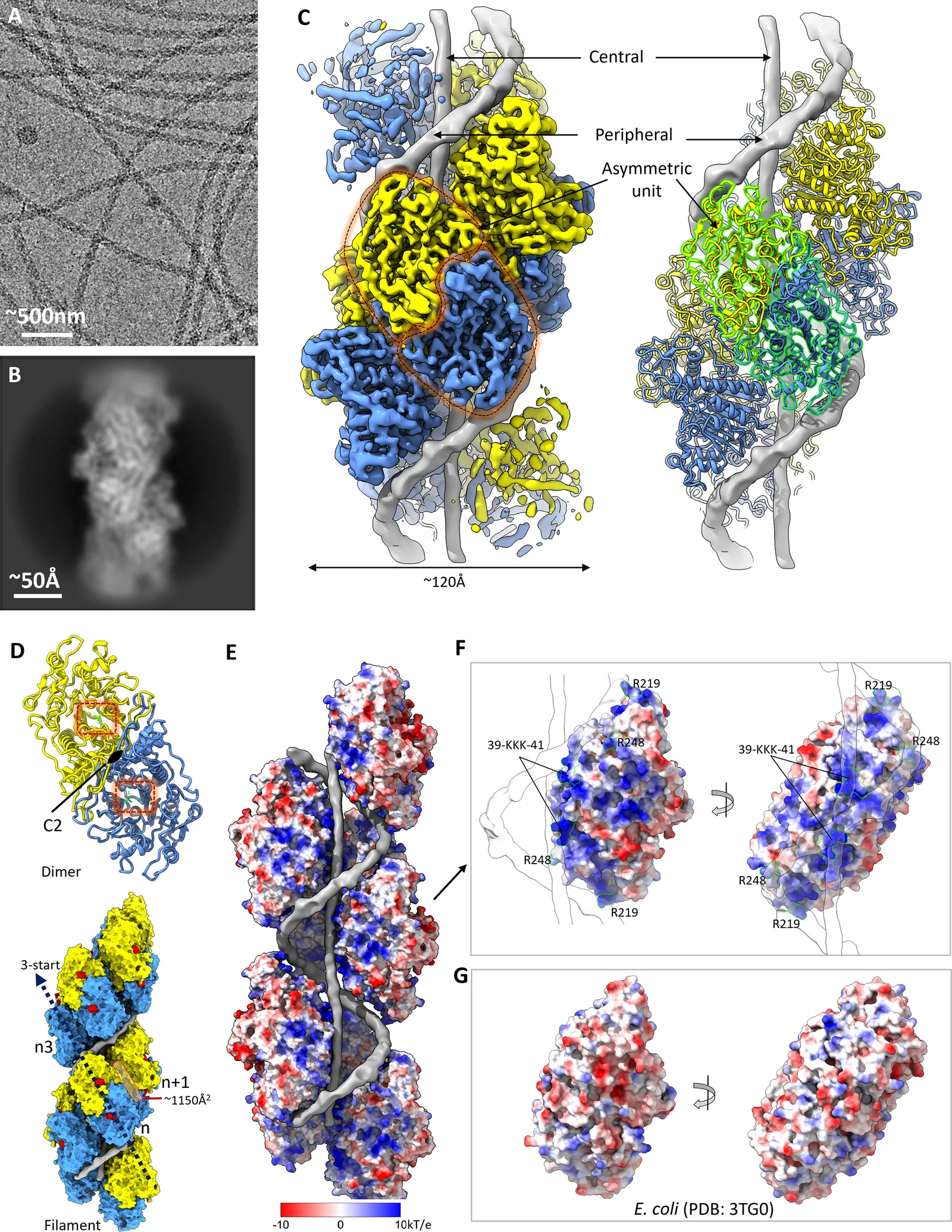
Cryo-EM structure of the filamentous alkaline phosphatase (ALP) from *Pseudomonas aeruginosa*. (**A**) Representative cryo-EM micrograph and (**B**) 2D class average of the ALP filament. (**C**) Cryo-EM density map and corresponding atomic model illustrating the overall helical packaging of the asymmetric unit, ALP dimer, and the two unassigned (central and peripheral) densities in gray. (**D**) Spatial arrangement of active sites within the ALP dimer and filament assembly. (**E**) Cutaway view of the filament lumen highlighting the highly electropositive surface patches that contact the central and peripheral scaffolding densities. (**F**) Close-up view of a single ALP dimer within the filament, showing the 39-KKK-41 motif interacting with the central density and residues R219/R248 coordinating with the peripheral density. (**G**) Structure of the *E. coli* ALP dimer shown in an orientation identical to panel **F**, demonstrating the absence of comparable electrostatic patches corresponding to the 39-KKK-41, R219, and R248 residues of *P. aeruginosa* ALP.

Cryo-EM micrographs of an extracellular sheared sample from *Pseudomonas aeruginosa* grown under phosphate-limiting conditions revealed filaments of unknown identity, with an irregularly curved morphology and a helical architecture (Fig. 1A and B). Such irregular curvature resulted in a poor resolution when helical symmetry was imposed. To overcome this problem, we employed asymmetric reconstruction by relaxing symmetry constraints, a strategy successfully leveraged in prior challenging filament studies (9). This approach substantially improved the map, yielding a ~3.7 Å resolution density map that enabled unambiguous atomic model building and identified the filament-forming protein as ALP (NCBI RefSeq: WP_003112133.1) (Fig. 1C; Supplemental methods and Table S1).

The structure reveals that the filament is built from helically arranged ALP homodimers—the classical oligomeric state for this enzyme (10, 11). Tilted at ~40° relative to the central filament axis, these ALP dimers assemble into a 1-start helix with a rise of 28.4 Å and a twist of 83.2° per dimer. Local refinement focused on a single ALP dimer with imposed C2 symmetry improved the resolution to ~3.3 Å, providing additional confirmation of the ALP assignment. While the active site, subunit-subunit interface, and stabilizing pair of disulfide bonds within each dimer are quite similar to the classic *Escherichia coli* ALP prototype (10–12), the map revealed two striking, unassigned densities: one running directly down the central axis of the filament and another wrapping along the outer periphery (Fig. 1C).

Does polymerization modulate ALP catalytic activity through spatial organization? Within the filament, active sites of neighboring dimers are spaced ~62 Å apart—nearly double the internal ~35 Å distance between active sites within a single dimer (Fig. 1D). Notably, despite this dense molecular packing, all active sites remain fully exposed on the exterior surface of the filament, maintaining unhindered accessibility to the surrounding milieu. Whether this expanded spatial network acts to optimize substrate flux, suppress activity, or coordinate activity in the extracellular matrix remains an intriguing, open question.

What drives this enzyme to polymerize? Direct protein-protein contacts form 1-start inter-dimer interactions, with each dimer (*n*) contacting its 1-start helical mates (*n* – 1, *n* + 1) via a network of hydrogen bonds and salt bridges burying ~1,150 Å^2^ of surface area. However, the true structural anchors appear to be the unassigned central and peripheral densities. The central density acts as a continuous inner scaffolding that anchors all ALP dimers along the axis, while the peripheral density bridges dimers in the 3-start left-handed helix (i.e. *n*, *n* + 3, *n* + 6…) (Fig. 1D and E).

Remarkably, the ALP dimer surfaces contacting these unassigned densities are consistently electropositive. The 39-KKK-41 motif surrounds the central axis density, while residues R219 and R248 contact the peripheral density (Fig. 1F). Intriguingly, these positively charged residues are completely absent in the homologous periplasmic *E. coli* ALP (UniProt P00634), which shares 74% sequence identity (Fig. 1G). This suggests that the *P. aeruginosa* ALP surface may have specifically evolved to facilitate the filament assembly.

Such concentrated electropositive tracks indicate that these unknown densities represent negatively charged biomolecules—likely extracellular DNA, RNA, acidic exopolysaccharides, teichoic acid, membrane fragments, or polyphosphate—that are typically found as extracellular biomolecules. In the absence of such charge-complementary molecules, electrostatic repulsion between the ALP dimers would structurally prohibit such filament assembly. While the exact chemical identities of the bridging molecules in our structure await further experiments, our findings establish the *P. aeruginosa* ALP assembly as a scaffold-dependent class of enzymatic polymers (13, 14).

### The ProtK nanotube

**Fig 2:**
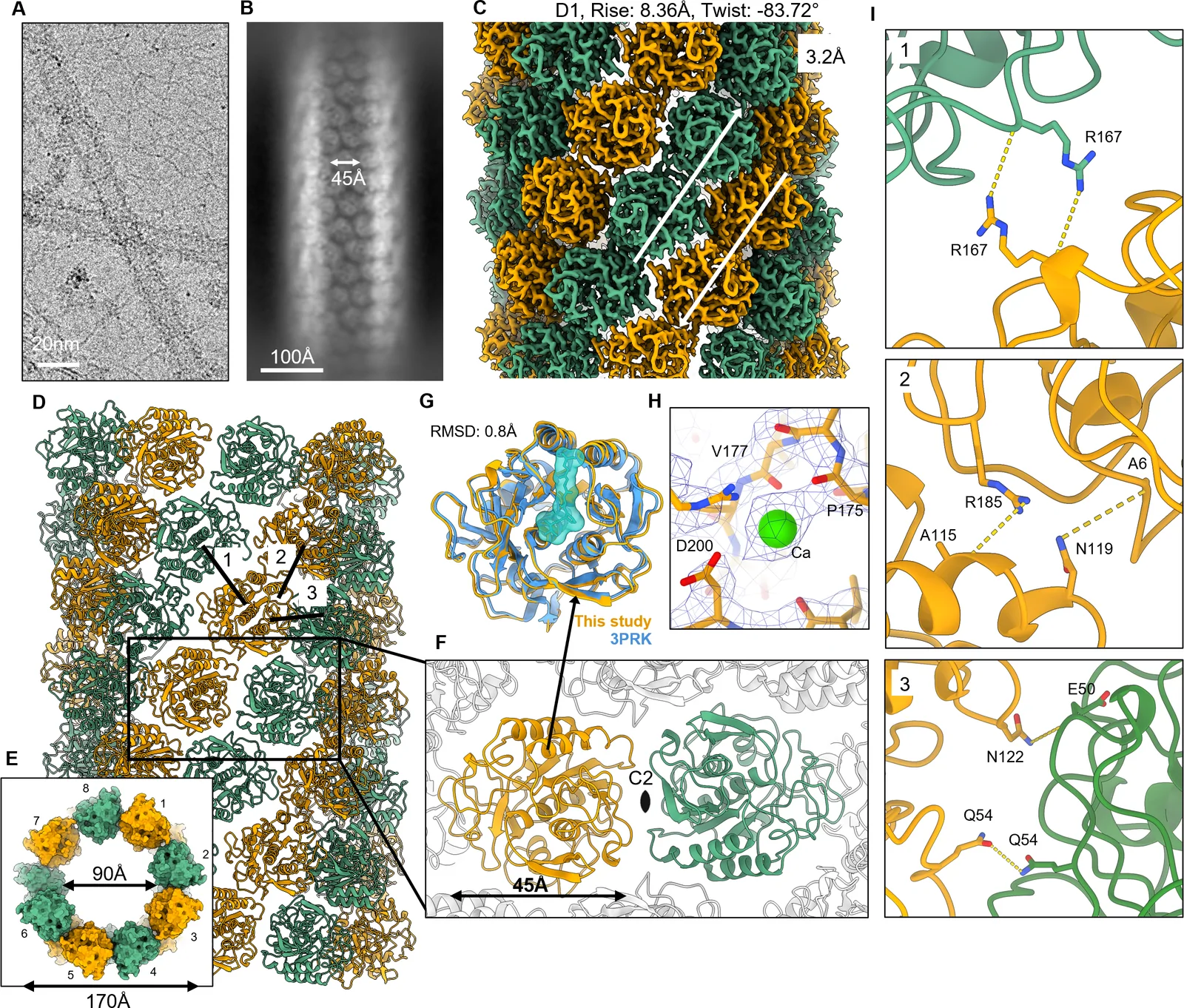
Cryo-EM structure of the proteinase K nanotube. (**A**) Representative cryo-EM micrograph of a proteinase K nanotube. Scale bar, ~20 nm; (**B**) 2D class average of nanotube segments. The inter-subunit distance (~45 Å) is indicated. Scale bar, ~100 Å; (**C**) Cryo-EM density map at 3.2 Å resolution, colored by alternating subunits (green and orange) to highlight the D1 symmetry. Helical parameters: rise = 8.36 Å, twist = −83.72°, D1 symmetry. (**D**) Cartoon representation of the atomic model. Black lines denote the three distinct inter-subunit contacts (1–3) described in panel **I**. (**E**) Cross-sectional view showing eight protofilaments. Outer diameter: ~170 Å, inner diameter: ~90 Å. (**F**) Enlarged view of the boxed region in panel **D** highlighting the inter-subunit local C2 symmetry axis (black diamond). The diameter of a single subunit is ~45 Å. (**G**) Superposition of the proteinase K monomer from the nanotube structure (orange) with the crystal structure 3PRK (blue, RMSD of 0.8 Å). 3PRK is a complex with a transition state analog (methoxysuccinyl-Ala-Ala-Pro-Ala-chloromethyl ketone, cyan density), used here to demonstrate that the active site is solvent accessible in the filament state; (**H**) Cryo-EM density (blue mesh) around the calcium-binding site, with the coordinating residues D200, P175, and V177 and the Ca²^+^ ion (green sphere) indicated. (**I**) Close-up views of the three inter-subunit interfaces numbered in panel **D**. Interface 1, symmetric R167–R167 hydrogen bonds; interface 2, hydrogen bonds involving R185–A115 and N119–A6; interface 3, contacts mediated by E50, N122, and Q54. Hydrogen bonds are shown as yellow dashed lines.

In a parallel study, we collected cryo-EM data on ProtK (commercial source: cat. no. 124568, Merck/Sigma-Aldrich) digested sacculi of the cable bacterium *Electrothrix gigas*. These micrographs revealed the presence of 17 nm diameter nanotubes of unknown composition (Fig. 2A and B). Standard helical processing was used to refine the rise and twist to 8.36 Å and −83.72° and yielded a 3.2 Å reconstruction (Fig. 2C), allowing for automated *de novo* model building using ModelAngelo (15), which identified the subunit as proteinase K (UniProt P06873, Fig. 2D; Supplemental methods and Table S1). The hollow tubes (with an inner diameter of ~90 Å) are composed of eight ProtK protofilaments that run in an antiparallel fashion as a result of the global dihedral (D1) symmetry (Fig. 2E and F).

A structural comparison of the ProtK monomer with the 3PRK crystal structure demonstrates that both are highly similar (RMSD across all 279 C_α_ atom pairs: 0.8 Å, Fig. 2G), indicating that the packing in the nanotube did not induce any structural rearrangements of the ProtK fold. As in the crystal structure, we see connected density between cys123 and cys34 and between cys178 and cys249. In addition to these two disulfide bonds, we also see density for a putative ion in the Ca-binding pocket (V177, P175, and D200) defined in 3PRK (Fig. 2H). Moreover, 3PRK is a complex with a transition state analog (methoxysuccinyl-Ala-Ala-Pro-Ala-chloromethyl ketone, cyan density) (Fig. 2G) (16). The structural overlay between 3PRK and our model (Fig. 2G) shows that the active site of ProtK is solvent accessible in the nanotube assembly (Fig. 2D through F).

To gain a deeper understanding of the driving forces behind ProtK polymerization, we analyzed the protomer packing interfaces. Based on this analysis, we identified three contact types (labeled as “1,” “2,” and “3” in Fig. 2D) that are highlighted in more detail in Fig. 2I. Contacts 1, 2, and 3 all involve the formation of just two hydrogen bonds, suggesting that the strengths of these binding interfaces are rather modest. We found no prior literature observations of ProtK oligomerization or filament formation, and an examination of the asymmetric units of the ProtK crystal structures (3PRK, 1IC6, 2ID8, 5WRC, 5B1E, 5AVJ, and 2HPZ) showed no indication of higher oligomers or crystal contacts involving the subunit interfaces observed in the ProtK filaments reported in this study. Each of the three inter-protomer contacts in the filaments is mediated by just two hydrogen bonds. Such minimal interfaces are more consistent with a concentration-driven packing than with a cooperatively regulated polymer. Accordingly, the ProtK nanotubes observed in this study may be an artifact of the elevated concentration of the stock solution (10 mg/mL in 0.5 mM Tris buffer, pH 8.9). Having said that, we did monitor for tube formation in the stock solution and found no evidence for the presence of ProtK nanotubes. It is not inconceivable that ProtK nanotube formation is triggered by a yet-to-be-identified component that is present in the cable bacteria sample. Regardless, it highlights that during biotechnological applications of ProtK, the enzyme can be sequestered into nanotubes, which, depending on the nature of the application, may be desirable (or not).

Could the proteinase K nanotube serve a biological purpose? There is no prior mention that ProtK adopts higher oligomeric forms in the native fungus *Tritirachium album*. Nevertheless, one cannot exclude that, under environmental conditions and high local concentrations reached upon secretion, ProtK self-association into a nanotube displaying outward-facing, catalytically competent active sites might buffer the enzyme against autolysis, serve as a labile storage depot, or present a high-avidity proteolytic surface. Moreover, nanotube formation could be under control of additional cooperative factors, as is observed in the *P. aeruginosa* ALP. Distinguishing an evolved function from an incidental property of a sticky, hyperstable enzyme will require direct tests of concentration dependence and of catalytic activity in the polymeric versus monomeric state. Either way, this study highlights that high-resolution imaging and 3D reconstruction directly from crude cell extracts can reveal unknown oligomeric forms of commonly used or studied enzymes, where such oligomerization may influence the physiological or biotechnological function of the enzymes involved.

## Supplementary Material

Reviewer comments

## Data Availability

The atomic models and maps for the alkaline phosphatase and proteinase K filaments have been deposited in the Protein Data Bank and the Electron Microscopy Data Bank with accession codes 36PY and EMD-77749 and 28EP and EMD-81689, respectively.

## SUPPLEMENTAL MATERIAL

Supplemental materials spectrum.02439-26-s0001.pdf Supplemental methods and Table S1.

OPEN PEER REVIEW reviewer-comments.pdf An accounting of the reviewer comments and feedback.

ASM does not own the copyrights to Supplemental Material that may be linked to, or accessed through, an article. The authors have granted ASM a non-exclusive, world-wide license to publish the Supplemental Material files. Please contact the corresponding author directly for reuse.
