# Supplementary material for: Protein polymers proliferate: polymerization of phosphatases and proteases
Source: Microbiol Spectr. 2026 Sep 14;14(10):e02439-26. doi: 10.1128/spectrum.02439-26 (PMC13689332; doi:10.1128/spectrum.02439-26)
Supplement: Supplementary file 1 [file reviewer-comments.pdf]

ASM | Microbiology Spectrum

Protein polymers proliferate:polymerization of phosphatases and proteases

Ravi Sonani, Iman Chouikha, Thijs Bodson, Silvia Hidalgo-Martinez, Filip Meysman, Han Remaut, Geneviève Ball, Edward Egelman, Rome Voulhoux, and Mike Sleutel

Corresponding Author(s): Edward Egelman, University of Virginia School of Medicine

**Review Timeline:**		
	Submission Date:	July 15, 2026
	Editorial Decision:	July 28, 2026
	Revision Received:	July 29, 2026
	Accepted:	August 11, 2026

Editor: Emily Weinert

Reviewer(s): The reviewers have opted to remain anonymous.

Transaction Report:

(Note: With the exception of the correction of typographical or spelling errors that could be a source of ambiguity, letters and reports are not edited. The original formatting of letters and referee reports may not be reflected in this compilation.)

DOI: https://doi.org/10.1128/spectrum.02439-26

Re: Spectrum02439-26 (**Protein polymers proliferate:polymerization of phosphatases and proteases**)

Dear Dr. Egelman:

Thank you for the privilege of reviewing your work. I am pleased to inform you that your manuscript has been editorially accepted for publication. However, there are a few additional questions in the submission form that need to be answered before your manuscript is officially accepted. Once these are completed, please return your submission so that I can move your paper forward to acceptance.

Please return the manuscript within 60 days; if you cannot complete the submission questions within this time period, please contact me.

Thank you for submitting your paper to Spectrum.

Sincerely,
Emily Weinert
Editor
Microbiology Spectrum

Reviewer# 1

I have only a few minor comments:

1. The unknown density in the ALP filament is suggested to be possibly be single stranded DNA or RNA. How similar are the helical parameters of the helical mystery density to those of single stranded DNA or RNA? Also, there isn't any apparent density for the DNA or RNA bases or sugars, but could those be absent due to disorder or averaging in the reconstruction analysis?

Due to the poor local resolution (>6 Å; see the local resolution map below), no discernible symmetry-related features are present within the unassigned density, preventing meaningful comparison with features of DNA or RNA. Further, single stranded DNA and RNA have no helical structure, so there is no "signature" to find. The limited resolution also precludes visualization of characteristic structural features such as nucleobases or sugar moieties. Indeed, the poor local resolution suggests that the associated molecule is likely conformationally heterogeneous or disordered. Further, averaging of molecules that are not in register could have led to the loss of most features. Although the dimensions of the density are broadly compatible with polyphosphate or the phosphate backbone of single-stranded DNA or RNA, we refrain from expanding this discussion in the manuscript because any assignment would be highly speculative in the absence of higher-resolution structural information.

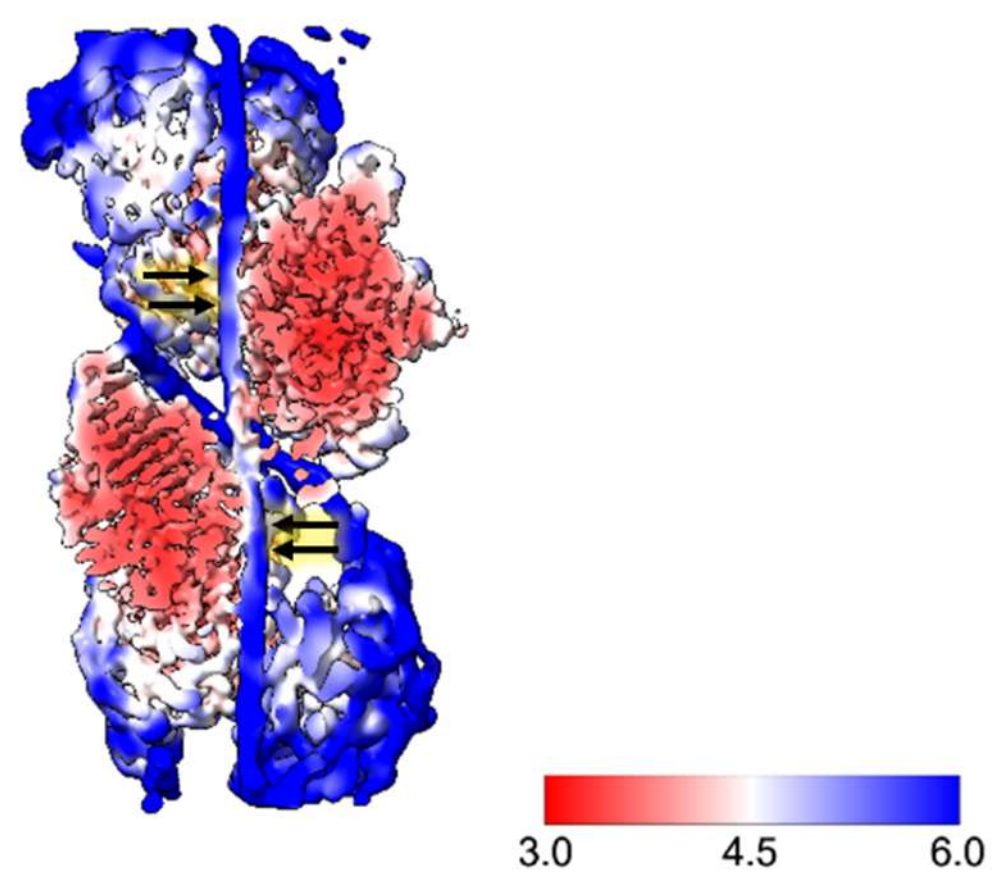

2. Can the authors think of any reason why ALP would interact with DNA, RNA, acidic exopolysaccharaides, teichoic acid, membrane fragments or polyphosphate? It may not be required to speculate on this in this short observation report, but it would be of interest to the reader.

There are several reasons why we consider these to be plausible candidates. First, all are highly negatively charged molecules, making them compatible with the strongly electropositive surfaces lining the ALP filament. Second, they are commonly found in the extracellular milieu where the filaments were observed. Third, despite the apparent disorder, their overall dimensions are broadly consistent with the size of the unassigned densities. In particular,

extracellular DNA and polyphosphate are attractive candidates because both can serve as extracellular phosphate reservoirs - potential substrates for ALP. Nevertheless, we emphasize that there is currently no direct experimental evidence supporting any specific molecular identity or interaction, and we therefore refrain from making a definitive assignment in this very short communication.

3. On page 4, at the end of the ALP section, an SgrAI paper is referenced as a scaffold-dependent class of enzymatic polymers. This may be because the SgrAI filaments depend upon DNA binding for filamentation, however, the DNA does not form a scaffold in the filament in the usual sense. Instead, SgrAI binds to specific target sequences in DNA, and the SgrAI/DNA complex then assembles into a filament. The DNA just outside of the target sequences is involved in interactions which stabilize the filament via protomer- protomer interactions, but the DNA need not be contiguous for this to occur. All SgrAI filament structures have been determined with relatively short DNA (40 bp) and with one molecule of DNA bound to one SgrAI dimer. Suggesting that it is a scaffold dependent filament gives the impression that the DNA runs along the helical axis and is contiguous which is not the case (instead it runs nearly perpendicular to the helical axis). The L7TIR plant immune receptor bound to nucleic acid (modeled as DNA in the structure, PDB file 7X5K) by Yu et al. 2022 (PMID: 35597242) may be a better example of what the authors would consider a scaffolded filament.

We appreciate the reviewer's perspective, but we believe that the definition of a scaffold-dependent polymer can be broader than one in which the scaffold passes along the helical axis. For example, a scaffold on a building under construction or repair is on the *outside* of the structure! We agree that using the relatively short SgrAI filament as an example could be confusing in the present context. We have therefore revised the text to remove SgrAI as an example of a scaffold-dependent polymer and instead cite the suggested study, which more closely fits the context here.

4. Page 3, first sentence: “Acetyl” need not be captalized.

Corrected.

5. Page 5, first paragraph, last sentence. I would change “could be (un)desirable” to “may be desirable (or not).”

Corrected.

6. Legend to figure 1, panel B. The legend indicates that there are multiple 2D class averages but only one is shown.

Thank you for catching this. Corrected.

7. Legend to figure 2, panel B. The legend indicates that there is an inset, but I do not see one.

Thank you for catching this. Corrected.

Reviewer# 2

I have just some minor comments and suggestions:

1) Regarding the conditions in which the alkaline phosphatase filaments were observed – phosphate-starved P. aeruginosa - can the authors speculate about what these changes in phosphate levels may do to the bacterium's physiology? I immediately wondered whether cells generate more polyphosphate under those conditions, which might explain the scaffolding densities in this structures. Could the authors also comment on the approximate diameter of the structures – is there sufficient space to fit nucleic acids, or can those be ruled out on the basis of size (the central axis and peripheral grooves look like they may be too small to accommodate RNA or ssDNA?).

Under phosphate-limiting conditions, *P. aeruginosa* experiences phosphate starvation, and one attractive hypothesis is that ALP filamentation could enhance phosphate scavenging by increasing the availability of hydrolyzed inorganic phosphate. However, because we have not yet experimentally determined whether filament formation enhances or suppresses ALP activity, the proposed functional role remains speculative at this stage.

The limited local resolution (>6 Å; see the local resolution map above) suggests that the associated density is conformationally heterogeneous or disordered, or that averaging has eliminated features that are not in register. Although the dimensions of the density are broadly compatible with polyphosphate or the phosphate backbone of single-stranded DNA or RNA, this interpretation assumes that the nucleobases are sufficiently flexible or disordered that they are not seen in the reconstruction, or averaged out due to lack of register. The ALP filament has an outer diameter of approximately 120 Å and a central pore of ~10 Å. While a complete ssDNA or ssRNA strand would not fit within a uniform 10 Å channel, the central density occupies a staggered cavity that widens on alternating sides of the filament, creating additional space that could potentially accommodate nucleobases (see black arrows in the figure). Thus, based on size alone, neither single-stranded nucleic acids nor polyphosphate can be excluded as candidates for the unidentified density. Nevertheless, given the limited local resolution and the absence of diagnostic structural features, we believe that assigning a specific molecular identity would be premature and therefore refrain from further speculation in the manuscript.

2) The alkaline phosphatase filament is described as irregular, which is unclear. Does this just mean that they ended up having poor helical symmetry (as they go on to describe), or do the authors mean something else? Please clarify.

We have revised the text to clarify that "irregular" refers specifically to the irregularly curved nature of the filaments. This structural flexibility and deviation from a straight axis leads to poor results when helical symmetry is imposed. We have revised the manuscript to clarify this description.

3) The authors might consider processing the alkaline phosphatase structure using the D1 symmetry of the dimer in the asymmetric unit (or I suppose it could be processed as C2) rather than C1 - even without helical symmetry imposition, the two-fold symmetry should increase resolution, unless there is clear asymmetry that cautions against imposing that symmetry?

We thank reviewer for this suggestion. By enforcing local C2 symmetry within an ALP dimer, we improved the resolution to ~3.3 Å. This higher resolution map provides strong structural evidence that confirms our ALP assignment with high confidence. These results have been integrated into the revised manuscript.

4) What species is the commercial proteinase K from? Is it T. album? It would be good to clarify this.

Yes, it was from *T. album*. We have clarified this in the revised Supplement.

Re: Spectrum02439-26R1 (**Protein polymers proliferate:polymerization of phosphatases and proteases**)

Dear Dr. Egelman:

Your manuscript has been accepted, and I am forwarding it to the ASM production staff for publication. Your paper will first be checked to make sure all elements meet the technical requirements. ASM staff will contact you if anything needs to be revised before copyediting and production can begin. Otherwise, you will be notified when your proofs are ready to be viewed.

Data Availability: ASM policy requires that data be available to the public upon online posting of the article, so please verify all links to sequence records, if present, and make sure that each number retrieves the full record of the data. If a new accession number is not linked or a link is broken, provide production staff with the correct URL for the record. If the accession numbers for new data are not publicly accessible before the expected online posting of the article, publication may be delayed; please contact ASM production staff immediately with the expected release date.

Publication Fees: For information on publication fees and which article types have charges, please visit our website. We have partnered with Copyright Clearance Center (CCC) to collect author charges. If fees apply to your paper, you will receive a message from no-reply@copyright.com with further instructions. For questions related to paying charges through RightsLink, please contact CCC at ASM_Support@copyright.com or toll free at +1-877-622-5543. CCC makes every attempt to respond to all emails within 24 hours.

ASM Membership: Corresponding authors may join or renew ASM membership to obtain discounts on publication fees. Need to upgrade your membership level? Please contact Customer Service at Service@asmusa.org.

Embargo Policy: A press release may be issued as soon as the manuscript is posted on the Spectrum Latest Articles webpage. The corresponding author will receive an email with the subject line "ASM Journals Author Services Notification" when the article is available online.

Cover Image Submissions: If you would like to submit a potential Cover Image, please email a file and a short legend to spectrum@asmusa.org. Please note that we can only consider images that (i) the authors created or own and (ii) have not been previously published. By submitting, you agree that the image can be used under the same terms as the published article. Image File requirements: TIF/EPS, 7.5 inches wide by 8.25 inches tall (at least 2,250 pixels wide by 2,475 pixels tall), minimum 300 dpi resolution (600 dpi preferred), RGB, and no figure elements, e.g., arrows or panel labels. The legend should be a short description of the image, 1-2 sentences recommended. Please use the ASM Journals Cover Image Sizing Tool (https://journals.asm.org/pb-assets/html/asm-cover-sizing-tool.html) to ensure that your proposed cover image meets our size requirements.

The ASM Journals program strives for constant improvement in our submission and publication process. Please tell us how we can improve your experience by taking this quick Author Survey.

Thank you for submitting your paper to Spectrum.

Sincerely,
Emily Weinert
Editor
Microbiology Spectrum
