# Supplementary material for: Protein polymers proliferate: polymerization of phosphatases and proteases
Source: Microbiol Spectr. 2026 Sep 14;14(10):e02439-26. doi: 10.1128/spectrum.02439-26 (PMC13689332; doi:10.1128/spectrum.02439-26)
Supplement: Supplementary file 2 [file spectrum.02439-26-s0001.pdf]

Supplementary Materials

Protein polymers proliferate: polymerization of phosphatases and proteases

Ravi R. Sonani[1], Iman Chouikha[2], Thijs Bodson[3,4], Silvia Hidalgo-Martinez[5], Filip Meysman[5], Han Remaut[3,4], Geneviève Ball[2], Edward H. Egelman[1,*], Romé Voulhoux[2,*], Mike Sleutel[3,4,*]

[1]Department of Biochemistry and Molecular Genetics, University of Virginia Medical School, Charlottesville, VA 22903, USA

[2]CNRS, Aix Marseille Université, LCB-UMR7283, IM2B, Marseille, France

[3]Structural Biology Brussels, Department for Bio-engineering Sciences, Vrije Universiteit Brussel, Brussels 1050, Belgium.

[4]Structural and Molecular Microbiology, Vlaams Instituut voor Biotechnology (VIB) Center for Structural Biology, Vrije Universiteit Brussels, Brussels 1050, Belgium.

[5]Department of Biology, University of Antwerp; Antwerp, Belgium.

*Correspondence to: egelman@virginia.edu, voulhoux@imm.cnrs.fr, mike.sleutel@vub.be

This supplementary material file contains:

- Methods
- Supplementary Table 1
- References list for Methods

Methods

Cryo-EM of alkaline phosphatase (ALP) filaments from *Pseudomonas aeruginosa*

***Grid preparation**:* A 3 µL aliquot of the extracellular filament suspension was deposited onto a glow-discharged Lacey carbon grid. Following sample application, the grids were blotted for 3.5 sec under 100% humidity at 20°C and rapidly vitrified in liquid-nitrogen-cooled liquid ethane using a Vitrobot Mark IV automatic plunge freezer. The vitrified grids were stored in liquid nitrogen until data collection.

Data collection: Preliminary evaluation of grid quality, sample density, and ice thickness was carried out using a 200kV Glacios electron microscope. The final dataset was acquired on a 300kV Titan Krios microscope operating with a BioQuantum energy filter and a Gatan K3 direct electron detector. Micrographs were recorded at magnification of 81,000x which corresponds to a physical pixel size of 1.065 Å. Over a target defocus range of -0.8 to -2.4 µm, movie series were collected (40 frames per movie) with a total exposure of 50 e^{-}/Å^2. Data collection was controlled automatically via the Thermo Fisher Scientific EPU software suite.

Image processing and reconstruction: Cryo-EM data processing was carried out in cryoSPARC (1). Micrographs were motion-corrected using Patch Motion Correction, and CTF estimation was performed with Patch CTF refinement. Filament segments were initially picked manually from a subset of micrographs to produce 2D templates, which were then used for automated template-based picking across the complete dataset. Filament picks were cleaned via 2D classification to remove poorly aligned and bad particles. An initial 3D volume was generated using Ab-initio reconstruction and refined by Homogeneous Refinement using picks in the best-resolving 2D classes. This map was further refined through iterative rounds of Non-uniform Refinement with Local CTF refinement ON. Local resolution filtering of the map yielded the final map with an overall resolution of ~3.7 Å (Map:Map FSC, threshold = 0.143).

Model Building and Refinement: An initial atomic model was generated directly from the cryo-EM map using ModelAngelo (2). A BLAST search of this model sequence against the *Pseudomonas aeruginosa* proteome identified the protein as alkaline phosphatase. AlphaFold(3) predicted model of alkaline phosphatase was docked into the density and refined through cycles of refinement in COOT (4), ISOLDE (5) and Phenix real-space refinement (6). Final model statistics are reported in Supplementary Table 1.

Cryo-EM of proteinase K (protK) nanotubes

***Sample preparation*:** Proteinase K fibers were formed during the second step of a two-step extraction process applied to cable bacteria. To this end, individual cable bacterium filaments

(*Electrothrix gigas*) (7) were isolated from a clonal culture using custom-made glass hooks. Filaments were washed under a binocular microscope by transferring them between droplets of Milli-Q water to remove attached organic material and mineral particles. In a first step, these filaments were subjected to a chemical extraction procedure to remove lipid membranes and cytoplasmic content, yielding a so-called "fiber skeleton" (as described in detail in (8)). To this end, filaments were first incubated for 10 min in a droplet containing 1% (w/w) sodium dodecyl sulfate (SDS). Following lysis, filaments were washed in Milli-Q water droplets (6 x) to remove cellular debris. Subsequently, filaments were incubated for 10 min in 1 mM ethylenediaminetetraacetic acid (EDTA; pH 8.0) to remove residual SDS. Finally, the filaments were washed again in Milli-Q water droplets, thus resulting in fiber skeletons.

To further degrade the fiber skeletons, an enzymatic digestion was applied using proteinase K (from *Tritirachium album*; cat. no. 124568, Merck/Sigma-Aldrich, Darmstadt, Germany). A proteinase K stock solution (10 mg/mL) was diluted to 100 µg/mL in 0.5 mM Tris buffer (pH 8.9). The working solution was subsequently diluted to a final concentration of 10 µg/mL in 0.5 mM Tris buffer (pH 8.9) containing purified fiber skeletons. Samples were incubated for 90 min at 42 °C. Following digestion, samples were centrifuged for 5 min at 14,000 × g and the supernatant was removed. Fresh Tris buffer (0.5 mM, pH 8.9) was added and the centrifugation step was repeated three times to remove residual proteinase K.

Grid preparation: The resulting sample (centrifuged pellet redissolved in Tris buffer) was used cryo sample peparation using a Leica GP2 automated plunge freezer (Leica Microsystems, Vienna, Austria) operated at 20 °C and 10% relative humidity. Quantifoil R2/1 300-mesh copper holey carbon grids were used, which were glow-discharged for 30 s at a plasma current of 5 mA using an ELMO glow discharger (Agar Scientific). Before plunging, n-dodecyl β-D-maltoside (DDM) was added to the sample to 0.1% (w/v) final concentration. A 3 µL aliquot of the sample was applied to the TEM grid, back-blotted for 3.5 s using Whatman Grade 2 filter paper, and plunge-frozen into liquid ethane cooled to −186 °C.

Data collection: High-resolution cryo-EM images and movies were acquired on a JEOL CryoARM300 transmission electron microscope operated at 300 kV and equipped with an in-column Ω energy filter (12 eV slit width). Automated data acquisition was performed using SerialEM (v 4.1.8) (9). Images were recorded on a K3 direct electron detector operating in counting mode at a nominal magnification of x 60,000, corresponding to a calibrated pixel size of 0.71 Å. A total dose of 60 e^{-} $Å^{2}$ was fractionated over 60 movie frames with a defocus range from -1.2 to -2.0µm.

Image processing and reconstruction: Cryo-EM data processing was carried out in cryoSPARC (1). Micrographs were motion-corrected using Patch Motion Correction, and CTF estimation was

performed with Patch CTF refinement. Filament segments were picked with the Filament tracer and picks were cleaned via 2D classification to remove poorly aligned and bad particles. Initial estimates of the helical symmetry (rise and twist) was done via Fourier Bessel indexing of power spectra produced by the Average Spectra job. Helical refinement using a 170 Å diameter cylinder as starting model yielded the final reconstruction with an overall resolution of 3.2 Å (Map:Map FSC, threshold = 0.143). EMready (10) post-processing was used for figure production.

Model Building and Refinement: An initial atomic model was generated directly from the cryo-EM map using ModelAngelo (2). A BLAST search of this model sequence against the RefSeq database identified the protein as proteinase K (UniProt # P06873). AlphaFold (3) predicted model of proteinase K was docked into the density and refined through cycles of refinement in COOT (4), and Phenix real-space refinement (6). Final model statistics are reported in Supplementary Table 1.

Supplementary Table 1. CryoEM and Refinement Statistics

**Parameter**	**Alkaline Phosphatase**	**Proteinase K**
**Data collection and processing**		
Voltage (kV)	300	300
Electron exposure (e^{-} Å^{-2})	50	60
Pixel size (Å)	1.065	0.71
Particle images (n)	561233	55526
Shift (pixel)	-	12
**Reconstruction method**	**Asymmetric reconstruction**<br>Point group – C1	**Helical reconstruction**<br>Symmetry parameters:<br>Point group – D1<br>Helical Rise – 8.36 Å<br>Helical twist – -83.72°
**Map resolution (Å)**		
Map:map FSC (0.143)	3.7	3.2
Model:map FSC (0.5)	3.9	3.3
d_{99}	4.0	3.5
**Refinement and Model validation**		
Ramachandran Favored (%)	96.36	96.02
Ramachandran Outliers (%)	0.0	0.0
RSCC	0.84	0.90
Clash score	2.86	3.07
Bonds RMSD, length (Å)	0.013	0.002
Bonds RMSD, angles (°)	0.895	0.483
**Deposition ID**		
PDB (model)	36PY	28EP
EMDB (map)	EMD-77749	EMD-81689

References

1. Punjani A, Rubinstein JL, Fleet DJ, Brubaker MA. 2017. cryoSPARC: algorithms for rapid unsupervised cryo-EM structure determination. Nature methods 14:290-296.
2. Jamali K, Käll L, Zhang R, Brown A, Kimanius D, Scheres SH. 2024. Automated model building and protein identification in cryo-EM maps. Nature 628:450-457.
3. Abramson J, Adler J, Dunger J, Evans R, Green T, Pritzel A, Ronneberger O, Willmore L, Ballard AJ, Bambrick J. 2024. Accurate structure prediction of biomolecular interactions with AlphaFold 3. Nature 630:493-500.
4. Emsley P, Lohkamp B, Scott WG, Cowtan K. 2010. Features and development of Coot. Biological crystallography 66:486-501.
5. Croll TI. 2018. ISOLDE: a physically realistic environment for model building into low-resolution electron-density maps. Biological Crystallography 74:519-530.
6. Afonine PV, Poon BK, Read RJ, Sobolev OV, Terwilliger TC, Urzhumtsev A, Adams PD. 2018. Real-space refinement in PHENIX for cryo-EM and crystallography. Biological Crystallography 74:531-544.
7. Geelhoed JS, Thorup CA, Bjerg JJ, Schreiber L, Nielsen LP, Schramm A, Meysman FJ, Marshall IP. 2023. Indications for a genetic basis for big bacteria and description of the giant cable bacterium *Candidatus Electrothrix* gigas sp. nov. Microbiology Spectrum 11:e00538-23.
8. Boschker HT, Cook PL, Polerecky L, Eachambadi RT, Lozano H, Hidalgo-Martinez S, Khalenkow D, Spampinato V, Claes N, Kundu P. 2021. Efficient long-range conduction in cable bacteria through nickel protein wires. Nature communications 12:3996.
9. Mastronarde DN. 2005. Automated electron microscope tomography using robust prediction of specimen movements. Journal of structural biology 152:36-51.
10. He J, Li T, Huang S. 2023. Improvement of cryo-EM maps by simultaneous local and non-local deep learning. Nat. Commun. 14, 3217.
